# Evaluation of the association between quantitative mammographic density and breast cancer occurred in different quadrants

**DOI:** 10.1186/s12885-017-3270-0

**Published:** 2017-04-17

**Authors:** Siwa Chan, Jeon-Hor Chen, Shunshan Li, Rita Chang, Darh-Cherng Yeh, Ruey-Feng Chang, Lee-Ren Yeh, Jessica Kwong, Min-Ying Su

**Affiliations:** 1 Graduate Institute of Biomedical Electronics and Bioinformatics, National Taiwan University, Taipei, Taiwan; 2 Department of Medical Imaging, Tzu Chi General Hospital, Taichung, Taiwan; 3 Department of Radiology, Taichung Veterans General Hospital, Taichung, Taiwan; 4 Center for Functional Onco-Imaging, Department of Radiological Sciences, University of California, Irvine, CA USA; 5 Department of Radiology, E-Da Hospital and I-Shou University, Kaohsiung, Taiwan; 6 Breast Cancer Center, Tzu Chi General Hospital, Taichung, Taiwan; 7 John Tu and Thomas Yuen Center for Functional Onco-Imaging, University of California Irvine, No. 164, Irvine Hall, Irvine, CA 92697-5020 USA

**Keywords:** Mammographic density, Breast cancer, Breast quadrant, Dense area, Percent density, Upper-outer quadrant

## Abstract

**Background:**

To investigate the relationship between mammographic density measured in four quadrants of a breast with the location of the occurred cancer.

**Methods:**

One hundred and ten women diagnosed with unilateral breast cancer that could be determined in one specific breast quadrant were retrospectively studied. Women with previous cancer/breast surgery were excluded. The craniocaudal (CC) and mediolateral oblique (MLO) mammography of the contralateral normal breast were used to separate a breast into 4 quadrants: Upper-Outer (UO), Upper-Inner (UI), Lower-Outer (LO), and Lower-Inner (LI). The breast area (BA), dense area (DA), and percent density (PD) in each quadrant were measured by using the fuzzy-C-means segmentation. The BA, DA, and PD were compared between patients who had cancer occurring in different quadrants.

**Results:**

The upper-outer quadrant had the highest BA (37 ± 15 cm^2^) and DA (7.1 ± 2.9 cm^2^), with PD = 20.0 ± 5.8%. The order of BA and DA in the 4 separated quadrants were: UO > UI > LO > LI, and almost all pair-wise comparisons showed significant differences. For tumor location, 67 women (60.9%) had tumor in UO, 16 (14.5%) in UI, 7 (6.4%) in LO, and 20 (18.2%) in LI quadrant, respectively. The estimated odds and the 95% confidence limits of tumor development in the UO, UI, LO and LI quadrants were 1.56 (1.06, 2.29), 0.17 (0.10, 0.29), 0.07 (0.03, 0.15), and 0.22 (0.14, 0.36), respectively. In these 4 groups of women, the order of quadrant BA and DA were all the same (UO > UI > LO > LI), and there was no significant difference in BA, DA or PD among them (all *p* > 0.05).

**Conclusions:**

Breast cancer was most likely to occur in the UO quadrant, which was also the quadrant with highest BA and DA; but for women with tumors in other quadrants, the density in that quadrant was not the highest. Therefore, there was no direct association between quadrant density and tumor occurrence.

**Electronic supplementary material:**

The online version of this article (doi:10.1186/s12885-017-3270-0) contains supplementary material, which is available to authorized users.

## Background

The breast tissue mainly consists of two components: fibroglandular tissue and adipose tissue (fat). Fibroglandular tissue is a mixture of fibrous stroma and epithelial cells that line the ducts of the breast. Breast density measured by mammography (MD) is associated with the amount of fibroglandular tissue. Studies of mammographically dense tissues suggest that density may represent increased epithelial cellular concentration, stromal fibrosis, and epithelial hyperplasia [1]. MD has been proven as an independent risk factor for BC [2–9]. Women with dense tissue visible on a mammogram have a cancer risk 1.8 to 6.0 times that of women with little density [10]. The biological basis for higher cancer risk associated with increased MD is not fully understood. The cellular, biological, and genetic basis of the association between fibroglandular tissue and cancer risk were investigated in many studies, as described in detail in two review articles [11, 12]. MD was influenced by hormones and growth factors, and it was hypothesized that the combined effects of cell proliferation (mitogenesis) and genetic damage by mutagens (mutagenesis) led to the increased cancer risk [11]. The stroma composed of extracellular matrix proteins, adipocytes, fibroblasts and immune cells is also known to contribute to the increased cancer risk [12]. The strong evidence has led to a substantial effort to incorporate breast density into risk prediction models to improve accuracy [13–19].

A fundamental question that has yet to be answered is whether cancers tend to arise in mammographically dense tissue. Among few studies exploring the question, two studies showed that ductal carcinoma in situ (DCIS) [20] and invasive cancer [21] occurred overwhelmingly in the mammographically dense areas, suggesting that some aspects of glandular/stromal tissue comprising the dense tissue directly influences the carcinogenic process. Another study, however, found that after accounting for the overall percent density (PD) differences, density in the region was not a significant risk factor associated with the location of subsequently developed cancer [3].

Many studies have shown that the upper outer quadrant of the breast is the most frequent site for occurrence of breast cancer [22–24]. A study [23] consisting of 746 consecutive breast core biopsies noted 62% of 349 malignant lesions (95% confidence interval 57-67%) arose from the UO quadrant. An adequate explanation for this asymmetric occurrence of breast cancer within the breast has never been established. Since density is a risk factor, it would be very interesting to investigate the relationship between quadrant density and the tumor occurring quadrant location. Although this question has been raised for a long time, there were few publications in this area, possibly because of the lack of a reliable method that can measure quantitative density on mammography, as well as the lack of a standardized method that can divide a breast into four quadrants. The inconsistent results in a few published studies reporting quadrant or local breast density might also due to different methods that were used in the analysis [3, 20, 21]. Although many studies have reported the measurements of breast density using a variety of imaging modalities and methods, qualitatively or quantitatively, most studies analyzed the density in the whole breast, but not in well-defined quadrants.

In this work we applied a computer algorithm-based segmentation method to quantitatively analyze breast density on mammography, and also applied an established method to divide a breast into 4 quadrants based on craniocaudal (CC) and mediolateral oblique (MLO) mammography using the nipple and the chest wall muscle as references. A breast was separated into: upper-outer (UO), upper-inner (UI), lower-outer (LO), and lower-inner (LI) quadrants; and breast area (BA), dense area (DA) and PD in each quadrant were measured. For each woman, the occurrence of tumor in a specific quadrant was determined, and the women were separated into 4 groups that had tumors in UO, UI, LO, and LI quadrants, respectively. The presence of tumor would affect the measured density; therefore in this study we analyzed the quadrant density of the contralateral normal breast. Despite the fact that some degree of breast asymmetry was expected, the bilateral breasts were considered as symmetric in general, and the normal breast could be used to simulate the diseased breast before the tumor occurred [25–27]. After the women were separated into 4 groups based on tumor location, the BA, DA, and PD of 4 quadrants in the normal breasts of women in these 4 groups were compared to investigate the association of quadrant density with tumor location.

## Methods

### Subjects

This study was approved by the institutional review board and complied with the Health Insurance Portability and Accountability Act. From July 2012 to April 2014, mammography results of 213 women with pathologically confirmed cancer, who had no previous cancer/breast surgery, was retrospectively reviewed. The following women were excluded in the analysis for this study: 1) women with bilateral breast cancer (*N* = 2); 2) women with unilateral breast cancer that occupied more than one quadrant or was located in the subareolar area (*N* = 39); 3) women for whom the tumor location could not be determined on mammography (*N* = 15); 4) women for whom imaging issues occurred, including lack of acquisition of CC or MLO views, insufficient imaging quality for analysis, or those for whom the breast was not fully included in either view (*N* = 57). In total, the remaining 110 women were studied (mean age 55 year-old, range 31-85).

### Mammographic density segmentation

**Fig. 1:**
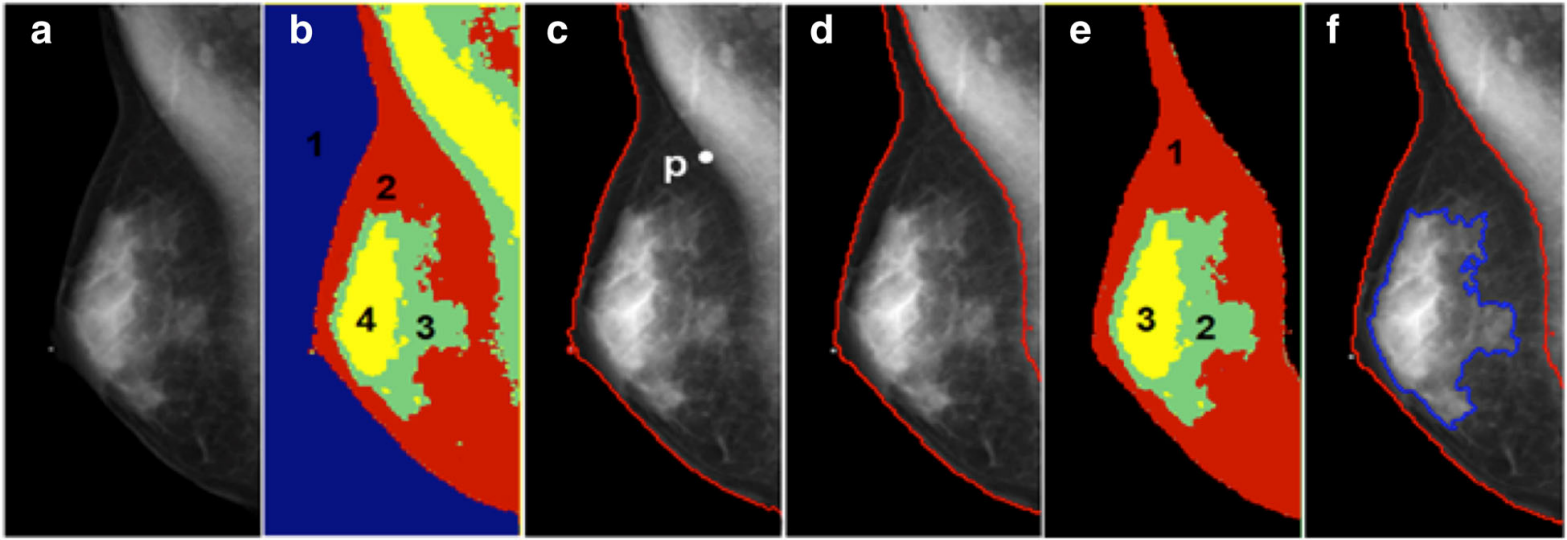
The quantification of mammographic density using Fuzzy C-means (FCM) segmentation method. **a** Original MLO mammogram. **b** Four FCM-clusters indicated by different colors. The cluster #1 is air and defines the anterior breast boundary. **c** A dividing point P on the breast-chest wall boundary is marked by the operator. **d** The breast-chest wall boundary is found after gradient tracing and b-spline curve fitting. **e** Three FCM-clusters are classified within the breast. The cluster #1 (*red color*) is fat, and cluster #2 and #3 represent dense tissues. **f** The breast area and dense tissue area is measured to calculate the percent density

All mammography was performed using digital mammographic systems (MAMMOMAT Inspiration Siemens, Erlangen, Germany). The standard CC and MLO views were acquired. In this study, we used a Fuzzy C-means (FCM) segmentation method to quantify the breast density [28–30]. The step-by-step procedures were illustrated in Fig. 1. We used 4 FCM-cluster numbers to separate different tissues on the mammographic image. Cluster #1 was air and defined the anterior breast boundary. To define the breast-chest wall boundary, a dividing point P was first marked by the operator on the breast–chest wall muscle interface. The breast-chest wall boundary was then identified by using gradient tracing and b-spline curve fitting. Within the defined breast area, three FCM-clusters (#2, 3, and 4) were classified. The cluster #1 (red color) was fat, and cluster #2 and #3 represented dense tissues. Lastly, the BA and DA were measured, and the ratio of DA/BA was calculated as the PD.

### Quadrant density measurement

**Fig. 2:**
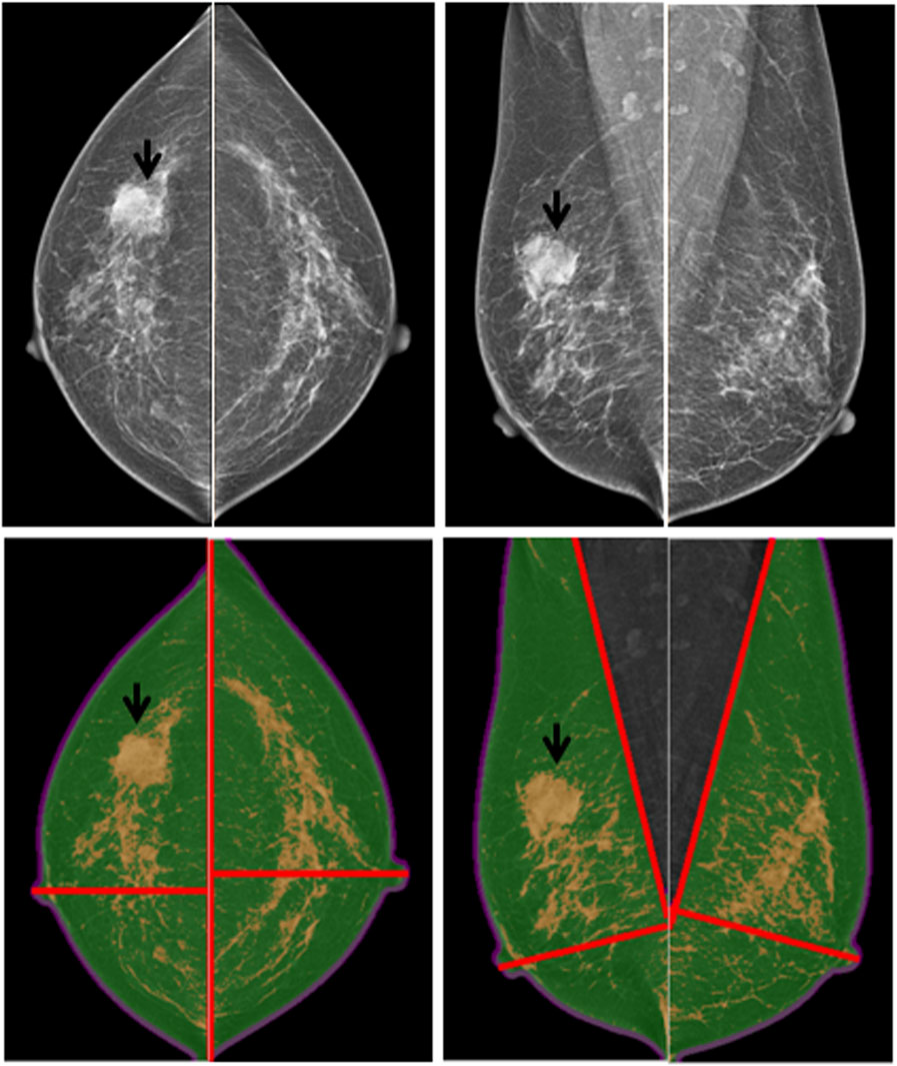
A woman with an invasive ductal carcinoma (*arrows*) in the UO quadrant of the right breast. *Upper panel*: original mammography. *Lower panel*: segmented breast area and density. Each view is divided into 2 partitions using a bisecting line through the nipple. In the left normal breast, the percent density is the highest in the UO quadrant (17.7%), followed by UI quadrant (15.2%), LO quadrant (14.2%), and LI quadrant (11.7%)

**Fig. 3:**
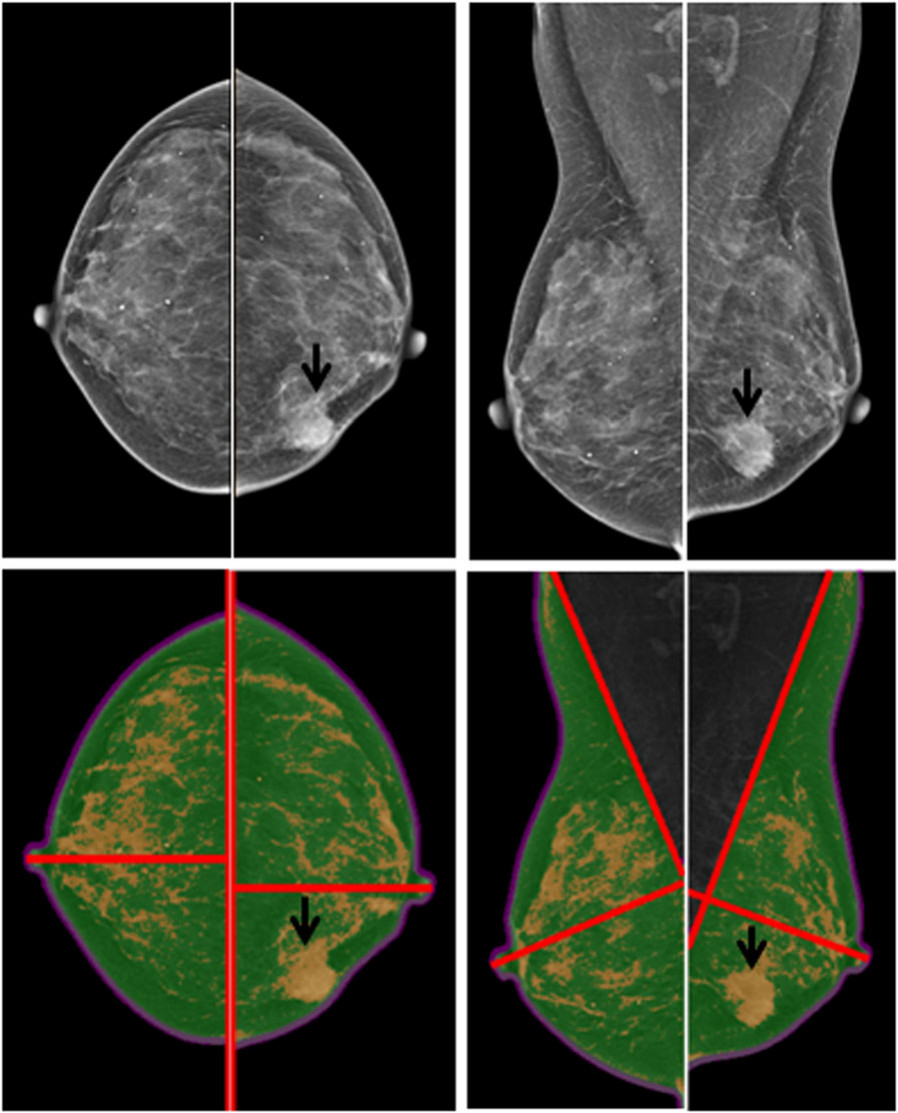
A woman with an invasive ductal carcinoma (*arrows*) in the LI quadrant of the left breast. *Upper panel*: original mammography. *Lower panel*: segmented breast area and density. Each view is divided into 2 partitions using a bisecting line through the nipple. In the right normal breast, the percent density is the highest in the UO quadrant (25.8%), followed by LO quadrant (22.2%), UI quadrant (18.7%), and LI quadrant (15.1%)

Quadrant separation was performed using the nipple and the chest wall boundary as anatomic landmarks to divide the breast into two partitions in each view, following the previously used dividing method [3, 20]. An automated algorithm was applied to divide the CC image into lateral and medial regions (i.e. CC-L and CC-M, respectively); and to divide the MLO image into superior and inferior regions (i.e. MLO-S and MLO-I, respectively) [3, 20]. Figures 2 and 3 show two case examples. For the CC view, the image edge was the chest wall, and the nipple location was manually defined. A bisecting line going through the nipple perpendicular to the image edge line was generated to separate “the medial region CC-M” and “the lateral region CC-L”. For the MLO view, a tangential line along the center of the chest wall boundary was used to define the edge line. Similarly as for the CC view, the nipple was manually defined and a bisecting line going through the nipple perpendicular to the breast-muscle line was generated to separate “the superior region MLO-S” and “the inferior region MLO-I”. The BA and DA in the separated CC-L, CC-M, MLO-S, and MLO-I were measured, and then were used to calculate the BA and DA for the four breast quadrants [3, 20]. The UO is the average of the CC-L and MLO-S; UI is the average of CC-M and MLO-S; LO is the average of CC-L and MLO-I; LI is the average of CC-M and MLO-I. Since the breast area was doubled counted in CC and MLO views, all measured results were divided by two to calculate the true area in cm^2^.

### Determination of tumor location in the four quadrants

The quadrant location of the breast cancer was determined using both CC and MLO views by an experienced radiologist (SC) who had 20 years of experience in interpreting mammography. Women with the following tumor characteristics were excluded from the study: bilateral tumors, tumors seen in more than one quadrant, tumors occurring in the subareolar region behind the nipple area and difficult to be assigned to one specific quadrant, and tumors unable to be clearly identified in the mammogram. In the remaining 110 women, they were separated into 4 groups with tumors in the UO, UI, LO, LI quadrants for statistical comparisons.

### Statistical considerations

The mean BA, DA, and PD in the four quadrants of all women were compared using paired student *t*-tests. The quadrants BA, DA, and PD among the 4 groups of women with tumors occurring in different quadrants were also compared using t-tests. Within each group of women who had a tumor in one quadrant only, the PD in the three other quadrants without visible tumor of the diseased breast and the corresponding three quadrants of the contralateral normal breast were compared using Pearson’s correlation coefficient to evaluate breast symmetry. To evaluate how the density in the tumor-occurring quadrant compared to the other 3 quadrants, they were ranked. The proportion of women who had the highest density in the tumor location quadrant was analyzed. If the density was associated with the tumor occurrence, the density of the tumor quadrant was expected to be the highest among all 4 quadrants, i.e. ranked as #1 among all 4 quadrants. Retrospectively, we also applied statistical method known as generalized estimating equations (GEE) to examine whether there was sufficient power to detect differences in the proportions of tumors among the 4 quadrants and between pairs of quadrants (Additional file 1).

## Results

### Patient characteristics

Of the 110 women included in this study, sixty-three women had a breast tumor in the left breast and forty-seven women had a breast tumor in the right breast. The cancer locations were pathologically proven, and the histological type included invasive ductal cancer (*N* = 77), invasive lobular cancer (*N* = 5), invasive mammary cancer of other types (*N* = 11), and ductal carcinoma in situ (*N* = 17). The tumor size was 2.1 ± 1.4 cm (mean ± STD) (range 0.1 cm – 7.0 cm). Three women had tumor larger than 5 cm (5.1 cm, 5.5 cm, and 7.0 cm).

### Breast area, dense area and percent density in four quadrants

**Fig. 4:**
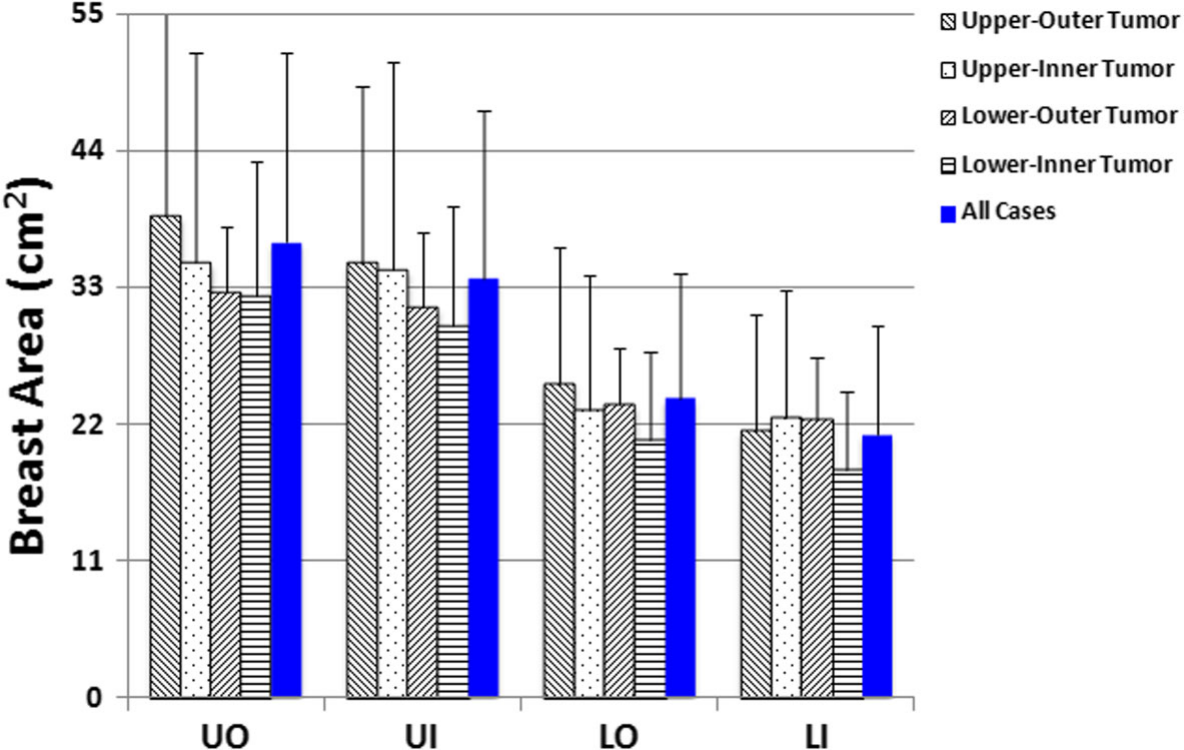
The *bar graph* showing the breast area in the four quadrants of the normal breast from all cases of 110 women, and from four groups of women with breast tumors in different quadrant locations. The order of BA is: UO > UI > LO > LI

**Fig. 5:**
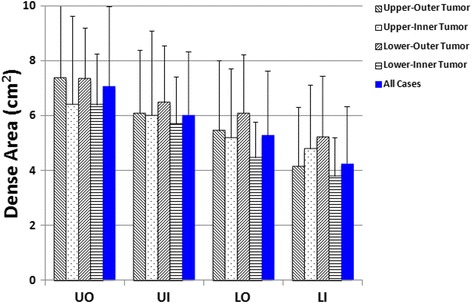
The *bar graph* showing the dense tissue area in the four quadrants of the normal breast from all cases of 110 women, and from four groups of women with breast tumors in different quadrant locations. The order of DA is: UO > UI > LO > LI

**Fig. 6:**
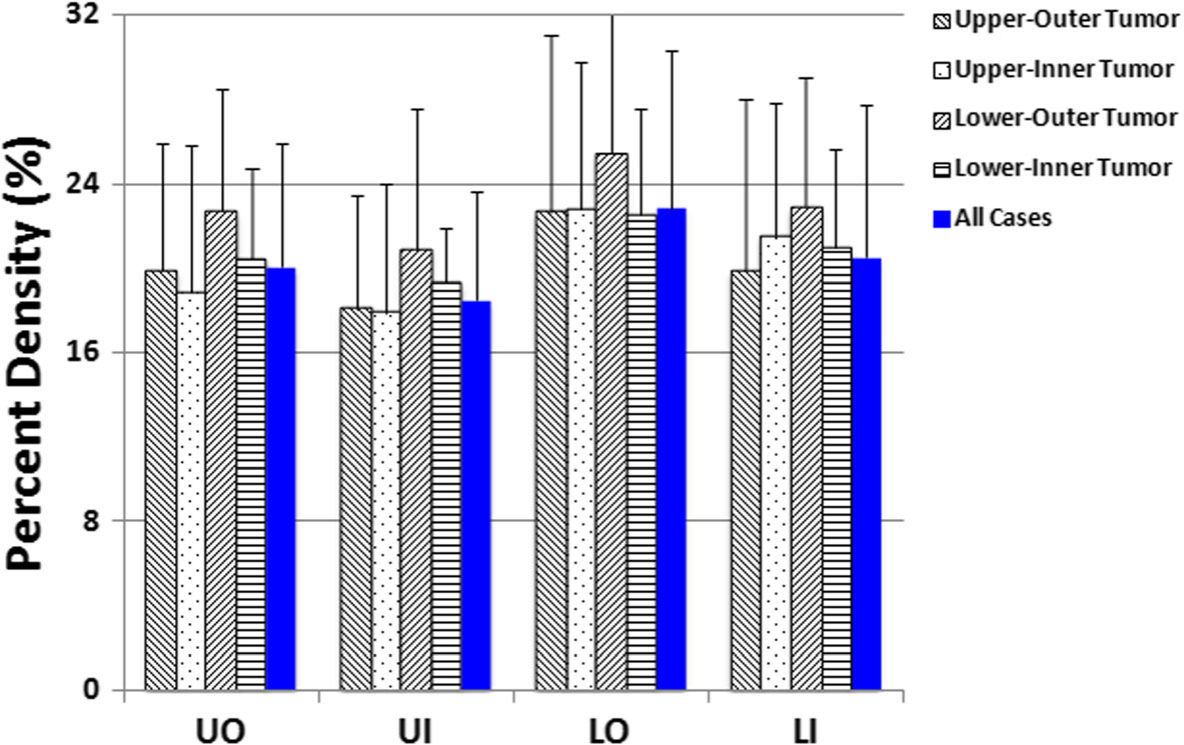
The *bar graph* showing the percent density in the four quadrants of the normal breast from all cases of 110 women, and from four groups of women with breast tumors in different quadrant locations. The order of PD is: LO > LI > UO > UI

In this study (*N* = 110), the mean overall PD in the contralateral normal breast was 20.2 ± 5.8%. Table 1 shows the BA, DA, and PD measured within the four quadrants of the normal breast from the 110 women. The UO quadrant had the highest BA with a mean ± standard deviation (SD) of 37 ± 15 cm^2^ and the highest DA (7.1 ± 2.9 cm^2^), with PD = 20.0 ± 5.8%. The order of BA in 4 quadrants was: UO > UI > LO > LI. The order of DA was exactly the same: UO > UI > LO > LI. The PD was calculated as the ratio of DA/BA, and the order was: LO > LI > UO > UI. The LO had the third ranking BA (24 ± 10 cm^2^) and DA (5.3 ± 2.4 cm^2^), but had the highest PD = 22.8 ± 7.5%. For each of the 110 women, except for the comparison of BA for UO vs. UI, and PD for UO vs. LI, pair-wise comparisons were significantly different (*p* < 0.05). Table 2 shows the mean + SD of BA, DA, and PD in the four quadrants for the four groups of women with tumors in different quadrants. In each group, the order of means for BA and DA were the following: UO > UI > LO > LI. The means for PD had the order of LO > LI > UO > UI. For each of the three variables, there was no significant difference between the 4 groups of women (*P* > 0.05 for all). Figure 4 shows a bar graph of BA in each of the 4 tumor groups and in the 110 cases. Figure 5 shows the results of DA, and Fig. 6 shows the results of PD.

**Table 1:** Breast area, dense area and percent density in the four quadrants and the whole breast (mean ± standard deviation from 110 cases)

	Breast area (cm^2^)	Dense area (cm^2^)	Percent density (%)
Upper-Outer (UO)	37 ± 15^a^	7.1 ± 2.9	20.0 ± 5.8^b^
Upper-Inner (UI)	34 ± 14^a^	6.0 ± 2.3	18.5 ± 5.2
Lower-Outer (LO)	24 ± 10	5.3 ± 2.4	22.8 ± 7.5
Lower-Inner (LI)	21 ± 9	4.3 ± 2.1	20.5 ± 7.3^b^
Whole Breast	116 ± 46	22.7 ± 8.8	20.2 ± 5.8

All pair-wise comparisons are significant with *p* < 0.05 except:

^a^The breast area in UO and UI are not significantly different

^b^The percent density in UO and LI are not significantly different

**Table 2:** Breast area, dense area and percent density in the four quadrants of four groups of women with tumors in different quadrants (mean ± standard deviation)

	UO tumor group	UI tumor group	LO tumor group	LI tumor group
Breast Area (cm^2^)				
Upper-Outer	39 ± 16	35 ± 17	33 ± 5	32 ± 11
Upper-Inner	35 ± 14	34 ± 17	31 ± 6	30 ± 10
Lower-Outer	25 ± 11	23 ± 11	24 ± 5	21 ± 7
Lower-Inner	22 ± 9	23 ± 10	22 ± 5	18 ± 6
Dense Area (cm^2^)				
Upper-Outer	7.4 ± 3.2	6.4 ± 3.2	7.4 ± 1.8	6.4 ± 1.9
Upper-Inner	6.1 ± 2.3	6.0 ± 3.1	6.5 ± 2.1	5.7 ± 1.7
Lower-Outer	5.5 ± 2.6	5.2 ± 2.5	6.1 ± 2.1	4.5 ± 1.3
Lower-Inner	4.2 ± 2.2	4.8 ± 2.3	5.2 ± 2.2	3.8 ± 1.4
Percent Density (%)				
Upper-Outer	19.9 ± 6.0	18.9 ± 7.0	22.7 ± 5.8	20.5 ± 4.3
Upper-Inner	18.1 ± 5.4	17.9 ± 6.1	20.9 ± 6.6	19.3 ± 2.5
Lower-Outer	22.7 ± 8.3	22.8 ± 7.0	25.5 ± 6.9	22.6 ± 5.0
Lower-Inner	19.9 ± 8.2	21.5 ± 6.3	22.9 ± 6.2	20.9 ± 4.7

### Tumor location and highest DA, PD in four quadrants

Table 3 shows the number of women with tumors in each of the UO, UI, LO, LI quadrants. Among the 110 women, 67 women (60.9%) had a tumor found in the UO, 16 women (14.5%) in the UI, 7 women (6.4%) in the LO, and 20 (18.2%) in the LI quadrant. Eighty-five women (77.3%) had the highest DA in the UO quadrant, and 47 women (42.7%) had the highest PD in the LO quadrant. This was consistent with the highest mean DA in the UO, and the highest mean PD in the LO. Fifty-eight women (58/110, 52.7%) had the tumor occurring in the highest DA quadrant (54 in UO, and 4 in UI). Thirty women (30/110, 27.3%) had the tumor occurring in the highest PD quadrant (21 in UO, 1 in UI, 3 in LO, and 5 in LI). We further investigated if there were associations between regional PD and the development of DCIS versus invasive carcinoma. We noted that only 3 of the 17 patients with DCIS (3/17 = 17.6%) and 27 of the 93 patients with invasive cancer (27/93 = 29.0%) had the tumor lesion in the breast quadrant with the highest PD. None of the two sub-cohorts showed the associations between regional PD and the development of breast cancer. Figure 2 shows a case example with a tumor that occurred in the upper outer quadrant that had the highest PD among those found in the 4 quadrants. Figure 3 shows another example with a tumor that occurred in the lower inner quadrant with the lowest PD.

**Table 3:** The number of women in the total of 110 who have tumor location and the highest DA and PD in four different quadrants

	Upper-Outer	Upper-Inner	Lower-Outer	Lower-Inner
Tumor location	**67 (60.9%)**	16 (14.5%)	7 (6.4%)	20 (18.2%)
Highest DA	**85 (77.3%)**	18 (16.4%)	4 (3.6%)	3 (2.7%)
Highest PD	33 (30.0%)	6 (5.5%)	**47 (42.7%)**	24 (21.8%)
Tumor in highest DA^a^	54	4	0	0
Tumor in highest PD^b^	21	1	3	5

^a^The total case number with tumor occurring in the quadrant with the highest DA is 54 + 4 = 58, (58/110 = 52.7%)

^b^The total case number with tumor occurring in the quadrant with the highest PD is 21 + 1 + 3 + 5 = 30, (30/110 = 27.3%)

The numbers in bold text represent the most frequent breast quadrants for tumor locations, highest DA, and highest PD respectively

### Association of tumor location with quadrant DA and PD

As shown above, the DA was the highest in the UO and also the tumors were the most likely to occur in the UO, which appeared to be related. However, this relationship might be simply due to that in the breast division the largest breast area and dense area was assigned to the UO. Therefore, further analysis was performed to assess whether this relationship also held for tumors occurring in other quadrants. We applied “ranking” to compare the DA and PD in the tumor occurring quadrant with the other three quadrants, and recorded the number of women (proportion) who had the ranking as #1, #2, #3, and #4. The results are shown in Table 4. In the 67 women with tumor in the UO, 54 women (80.6%) had the dense area in the UO ranked #1 (i.e. the highest among all 4 quadrants). In 16 women with tumor in UI, 8 (50%) had DA of UI ranked as #2. In 7 women with tumor in LO, 4 (57.1%) had the DA of LO ranked #3. Lastly in 20 women with tumor in LI, 14 (70%) had DA of LI ranked #4. Therefore, for most women in each group, the DA of the tumor occurring quadrant was following the trend of DA coming from quadrant separation (i.e. UO #1, UI #2, LO #3, LI #4). The PD was calculated by normalizing the DA with BA, and the results are also listed in Table 4. In majority of women with tumor occurring in UO (the highest and second highest proportion) the PD of UO ranked #2 and #3. Using a similar analysis, in the UI tumor group the PD of UI ranked #3 and #4; in the LO tumor group the PD of LO ranked #1 and #3; in the LI tumor group the PD of LI ranked #1 and #4. Therefore, the results showed that there was no trend, and PD was not associated with tumor occurrence.

**Table 4:** The number of woman whose dense area and percent density in the tumor occurring quadrant of the normal breast compared to the other three quadrants, shown as ranking

Group\Ranking	#1	#2	#3	#4
Dense Area				
UO Tumor (*N* = 67)	**54 (80.6%)** ^a^	9 (13.4%)	2 (3.0%)	2 (3.0%)
UI Tumor (*N* = 16)	4 (25%)	**8 (50%)** ^a^	4 (25%)	0 (0%)
LO Tumor (*N* = 7)	0 (0.0%)	2 (28.6%)	**4 (57.1%)** ^a^	1 (14.3%)
LI Tumor (*N* = 20)	0 (0%)	1 (5%)	5 (25%)	**14 (70%)** ^a^
Percent Density				
UO Tumor (*N* = 67)	13 (19.4%)	**23 (34.3%)** ^b^	**21 (31.4%)** ^b^	10 (14.9%)
UI Tumor (*N* = 16)	1 (6.2%)	0 (0.0%)	**7 (43.8%)** ^b^	**8 (50.0%)** ^b^
LO Tumor (*N* = 7)	**3 (42.9%)** ^b^	1 (14.2%)	**3 (42.9%)** ^b^	0 (0.0%)
LI Tumor (*N* = 20)	**7 (35%)** ^b^	4 (20%)	0 (0%)	**9 (45%)** ^b^

^a^Tumor in UO has the highest DA in UO, tumor in UI has the second highest DA in UI, tumor in LO has the third highest DA in LO, and tumor in LI has the lowest DA in LI. The order is consistent with the ranking of DA in 4 quadrants from the quadrant separation, i.e. UO > UI > LO > LI

^b^The ranking of the majority of women with the highest and the second highest percent density proportion in each breast quadrant. There is no trend

### Odds of tumor development within quadrants.

Based on GEE modeling, there was a statistically difference in the estimated odds of tumor development among the four quadrants (Score test, *p*-value <0.01). Significant differences were found between the proportions of tumors in pairs of quadrants, adjusted for multiple comparisons, indicating that the overall sample size and individual sample sizes in different quadrants provided sufficient power to compare proportions of tumors in the four quadrants (Additional file 2).

## Discussion

Although MD is associated with breast cancer risk, it is not known whether MD is directly related to cancer occurrence, i.e., tumors arising within the radiodense tissue [1]. Higher MD has, histologically, a greater cellular concentration and/or proliferation of the stroma or epithelium [20]. It was thus postulated that areas of higher density may be more susceptible to the initiation and promotion of breast cancers than areas of lower density [3]. Greater understanding of the association between density and cancer risk may provide information to improve the accuracy of cancer risk prediction and the clinical management of high-risk women [3]. Although many studies have investigated and demonstrated that mammographic density was an established risk factor, only a few studies evaluated the association of regional density with the location of the occurred cancer. Two studies measured breast density in different quadrants but showed inconsistent results for the correlation with the occurred cancer [3, 20]. Another study applied a computer algorithm to align serial images from the same woman, and used an overlaid grid analysis to measure density in 1-cm squares on prediagnostic mammographic films, and estimated the odds of subsequently developed tumor in relation to its prediagnostic square-specific MD [21]. The median prediagnostic MD was 98.2% (46.8%-100%) in 1-cm squares that subsequently contained the tumor, and 41.0% (31.5%-53.9%) in the whole breast [21]. The results suggested that tumors were more likely to occur in high MD areas.

In order to perform regional MD analysis, a standardized method to separate a breast into different regions (e.g. quadrants) as well as a reliable segmentation method that can yield quantitative density measurements were needed. In this study we applied a FCM algorithm to perform segmentation and quantify breast density in mammography. Different from Cumulus segmentation, which was focused on the outer boundary, the FCM segmentation was based on the pixel level. FCM could generate consistent results [31]; however, the segmentation was highly dependent on the choice of the total cluster number and the clusters that were assigned to differentiate between the dense and fatty tissue. Therefore, we tried to fix the cluster numbers used in the analysis. All final segmentation results were inspected by an experienced radiologist. When the segmentation results were unsatisfactory, the cluster numbers were adjusted. We have also implemented a previously reported method using a bisecting line through the nipple to separate the CC and MLO views into two partitions [3, 20]. The breast area and dense tissue area in the UO, UI, LO, and LI quadrants were calculated from the measurements in the CC view medial and lateral regions, and the MLO view superior and inferior regions.

**Fig. 7:**
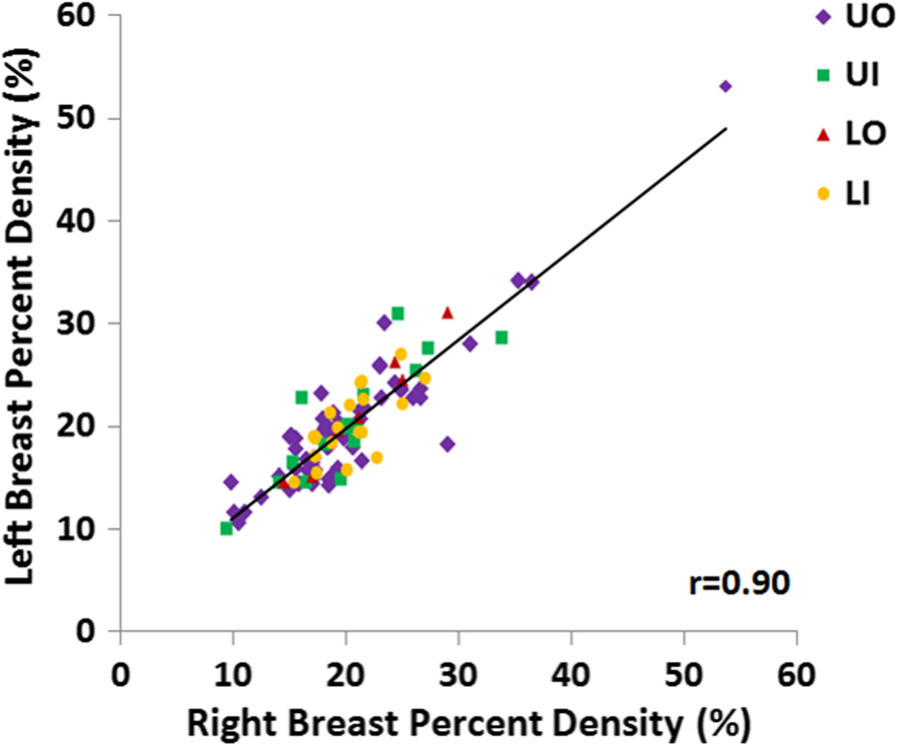
Correlation of the percent density in the three quadrants, which have no tumor, of the bilateral breasts. The tumor-quadrant is excluded. The four groups of women with tumor in different quadrants are shown by different symbols

Our datasets were from a cross-sectional study, and the women already had cancer in one breast. To overcome this problem, we analyzed the density in the contralateral normal breast, assuming that it mirrored the density in the diseased breast before the tumor occurred. In most women the bilateral breasts were in general symmetrical [25–27]. A study [25] to investigate the spatial distribution of density within the breast using 493 mammographic images from a sample of 165 premenopausal women showed that the degree of the spatial clustering of density was similar between a woman’s two breasts, and did not change with aging. We have also compared the measured density in the quadrants of the bilateral breasts which had no visible tumors, shown in Fig. 7. In each group of women with tumor in a specific quadrant, PD in the three quadrants which had no visible tumor was measured, and the results in the left and right breasts were compared. The Pearson correlation showed a strong correlation coefficient with *r* = 0.90, suggesting the validity of bilateral symmetry. If the tumor was very big, the presence of tumor might shift the density distribution or affect the separation of a breast into 4 quadrants in the diseased breast. The mean tumor size was 2.1 cm, relatively small within the mean breast area of 116 cm^2^. Without the presence of tumor, the left-right symmetry was expected to be better. The diseased breast was mainly used to determine the tumor location. The majority of presented results in this study were analyzed from the normal breast.

Given that the premise of the hypothesis in this study depended on bilateral breast symmetry, it would be preferable to analyze each patient’s symmetry utilizing more remote mammograms prior to the detectable cancer. Unfortunately we did not have that dataset in our current study, thus were unable to carry out the analysis. Overall, the assessment of symmetry in mammography is potentially limited by the fact that natural distortions between breasts are likely to occur during the course of breast compression routinely used in mammography. As such, symmetry measures can be confounded by the nature of the imaging procedure itself [26]. In our recently published results using 3D MRI in the study of breast density in 58 normal women, 47 pre-menopausal and 11 post-menopausal women [32], we found that bilateral breasts in women without cancer are highly symmetrical (*r* = 0.97 for breast volume, *r* = 0.97 for fibroglandular tissue volume, and *r* = 0.98 for PD). Another study using MRI showed small differences in the bilateral breast tissue composition, i.e. fat and water content, in young women and adults [33].

Our results showed that breast cancer was the most likely to occur in the UO quadrant (60.9%). This finding was consistent with most of the published studies in Western women [22–24], Eastern women [34], and Asian women [35, 36]. A study of Taiwanese women [18] showed that more than half (52.3%) of the primary breast tumors occurred in the UO quadrant. Other studies showed that the UO quadrant is also the most frequent location in many benign breast conditions including fibroadenoma and breast cysts [37], and phyllodes tumor [38]. The reasons why breast cancer occurs more frequently in the UO quadrant are not clear. One study reported that the high proportion of UO quadrant breast carcinomas was a reflection of the greater amount of breast tissue in this quadrant [23]. Another study found a disproportional annual increase in breast cancer in the UO quadrant, and that the proportion of UO quadrant breast cancer was the highest in the youngest age group [24], and it was postulated that the high rate of UO cancer might be related to the increasing use of cosmetics applied to the adjacent underarm and upper breast area. The underarm cosmetics are known to contain both DNA-damaging chemicals and chemicals which can mimic estrogen action [39], and the use of these cosmetics was reported to be associated with younger age for breast cancer diagnosis [40]. A recent study of genomic patterns of loss of heterozygosity and allelic imbalance in breast quadrants from 21 breast cancer patients showed increased levels of genomic instability in the outer breast quadrants, suggesting that a higher breast cancer rate in the UO quadrant might result from the development of genetic alterations in that region of the breast rather than from only a greater tissue volume [41]. These studies were mainly speculations, and so far there were few mechanistic studies published in the literature to investigate the etiology leading to the higher UO cancer occurrence rate.

Our results showed that among the four breast quadrants, the UO quadrant had the highest mean DA, and thus a larger amount of dense tissue might appear to be associated with the higher cancer rate in the UO. However, if the relationship between the amount of density tissue and cancer occurrence rate is true, the similar finding of higher density and higher cancer rate in the UO should be seen in cancers occurring in the other three quadrants as well. We first compared mean BA and DA among women with tumors occurring in the 4 quadrants. In each group the order was UO > UI > LO > LI. Also, after normalizing DA with BA, the order of the PD in each of the 4 groups was the same: LO > LI > UO > UI. Therefore, the quadrant area and density in these 4 groups of women were very similar, and the statistical analysis results showed that there was no significant difference between them. Then, we applied a “ranking” analysis method to investigate their relationship. For the DA, tumor in UI has the second highest DA in UI, tumor in LO has the third highest DA in LO, and tumor in LI has the lowest DA in LI. The order is exactly the same as the ranking of DA in 4 quadrants from the quadrant separation, i.e. UO > UI > LO > LI. For PD, the cancer-occurring quadrant had random rankings, and no trend at all. Only 30 of 110 women (27.3%) had cancer occurring in the quadrant with the highest PD. Therefore, there was no evidence to support that breast cancer was more likely to occur in the quadrant with the highest DA or PD. Our results concurred with a mammographic study [3] that regional breast density was not a significant risk factor for the subsequent development of breast cancer. Another study also concluded that a greater amount of breast tissue in a specific region could not solely explain the preference of breast cancer in the UO quadrant [24].

Besides quadrant PD, we also analyzed overall PD in the contralateral normal breast of the 110 women. Although in this study we did not have a matched case control group for the comparison, literature report on the comparison of MD between case and control groups in Asian women has shown significant difference in both pre- and post-menopausal women [42]. Two other studies, however, only showed significant density difference of the two groups in the postmenopausal women [43, 44].

This study had limitations. This was a small retrospective cross-sectional study and we analyzed the quadrant density from the contralateral normal breast to simulate the diseased breast before tumor occurred. A more convincing study design would have been to retrieve the prior mammogram of the diseased breast for analysis to predict the near future tumor occurrence. The density assessment on mammography was fundamentally limited by the fact that it was a 2D projection imaging method, and natural distortions between breasts were likely to occur during the breast compression. Although we have shown a strong left-right symmetry of *r* = 0.90 in the breast quadrants without tumor, some degree of left-right differences might come from the imaging procedures, not the intrinsic differences in breast tissues. New imaging modalities, such as digital breast tomosynthesis, may be more accurate in measuring proportion of glandular tissue by possibly reducing confounding factors, such as degree of compression and skin folds etc., in the measurement of breast density. Additional limitations are that the increased variance in parameter estimates for means and proportions depends on the relatively small overall sample size and the number of tumors detected in each quadrant and that the exclusion of individual patients was based on a variety of factors. However, we demonstrated that the sample sizes were sufficient to detect a statistically difference in the estimated odds of tumor development among the four quadrants. A single experienced radiologist provided interpretation of mammograms. It is possible that alternative interpretations would have been determined if additional experts were consulted. Additionally, confounding factors such as age, race/ethnicity, and diet, among others may have affected the results.

## Conclusions

In conclusion, in this study we used a computer-algorithm based segmentation method to quantify the dense tissue based on mammographic images, and also applied a standardized method to separate a breast into 4 quadrants. We found breast cancer was most likely to occur in the UO quadrant, which was also the quadrant with the highest BA and DA. However, for women with cancers occurring in UI, LO, or LI quadrant, the density in that quadrant was not the highest. When the breast area and density in the four groups of women with tumors occurring in four quadrants were compared, the results were very similar. All 4 groups of women showed the order of BA and DA as UO > UI > LO > LI, and the order of PD as LO > LI > UO > UI. Less than one quarter of women had breast cancer occurring in the quadrant with the highest PD. Our results showed that the differences in quadrant density was mainly from the breast division method, not related to the cancer occurrence. The amount of breast tissue in a specific quadrant cannot explain the preference of breast cancer occurring in a specific location.

## Additional files

Additional file 1: Generalized estimating equations (GEE). To examine whether there was sufficient power to detect differences in the proportions of tumors among the 4 quadrants and between pairs of quadrants. (DOCX 10 kb)

Additional file 2: Estimated odds of tumor development in the four quadrants. Based on GEE modeling, there was a statistically difference in the estimated odds of tumor development among the four quadrants. (DOCX 13 kb)

## Abbreviations

BA: Breast area
CC: Craniocaudal
DA: Dense area
DCIS: Ductal carcinoma in situ
FCM: Fuzzy C-means
LI: Lower-Inner
LO: Lower-Outer
MD: Mammographic density
MLO: Mediolateral oblique
PD: Percent density
UI: Upper-Inner
UO: Upper-Outer
