# Supplementary material for: Evaluation of the association between quantitative mammographic density and breast cancer occurred in different quadrants
Source: BMC Cancer. 2017 Apr 17;17:274. doi: 10.1186/s12885-017-3270-0 (PMC5392962; doi:10.1186/s12885-017-3270-0)
Supplement: Supplementary file 1 — Generalized estimating equations (GEE). To examine whether there was sufficient power to detect differences in the proportions of tumors among the 4 quadrants and between pairs of quadrants. (DOCX 10 kb) [file 12885_2017_3270_MOESM1_ESM.docx]

Because tumor development was examined within each quadrant for each individual, we applied a statistical method known as generalized estimating equations (GEE) to estimate and compare the odds of tumor development among four quadrants while taking into account the correlation among measurements within individual subjects. We specified a binomial model with a logit link function and an exchangeable correlation structure to estimate the odds of tumor development among four quadrants. The Bonferroni-Holm method was applied to maintain an overall significance level of 0.05 while adjusting for the multiple pairwise comparisons between quadrants.
