# Supplementary material for: Evaluation of the association between quantitative mammographic density and breast cancer occurred in different quadrants
Source: BMC Cancer. 2017 Apr 17;17:274. doi: 10.1186/s12885-017-3270-0 (PMC5392962; doi:10.1186/s12885-017-3270-0)
Supplement: Supplementary file 2 — Estimated odds of tumor development in the four quadrants. Based on GEE modeling, there was a statistically difference in the estimated odds of tumor development among the four quadrants. (DOCX 13 kb) [file 12885_2017_3270_MOESM2_ESM.docx]

As displayed in **Table S1**, the estimated odds and the 95% confidence limits of tumor development in the UO, UI, LO and LI quadrants were 1.56 (1.06, 2.29), 0.17 (0.10, 0.29), 0.07 (0.03, 0.15), and 0.22 (0.14, 0.36), respectively. **Table S2** gives the estimated odds ratios for the detection of tumors in pairs of quadrants. The odds of tumor development in the UO quadrant was 9.15 (95% 4.12, 20.33) times significantly higher than that of the UI quadrant, 22.93 (8.77, 59.94) times significantly higher than in LO quadrant, and 7.01 (3.23, 15.21) times significantly higher than in the LI quadrant. The odds of tumor development in the LO quadrant was reduced by 69% compared to the odds of tumor development in the upper-inner quadrant.

**Table S1**. **Estimated odds of tumor development in each of four quadrants from a GEE model**

| **Tumor Located Quadrant** | **Estimated Odds** | **95% Confidence Limit of the Estimated Odds** | |
| --- | --- | --- | --- |
| Upper-Outer | 1.56 | 1.06 | 2.29 |
| Upper-Inner | 0.17 | 0.10 | 0.29 |
| Lower-Outer | 0.07 | 0.03 | 0.15 |
| Lower-Inner | 0.22 | 0.14 | 0.36 |

**Table S2.** **Estimated odds ratios for the detection of tumors in pairs of quadrants**

| **Quadrants** | **Estimated Odds Ratio (OR)** | **95% Confidence Limits of the Estimated Odds Ratio (OR)** | |
| --- | --- | --- | --- |
| Upper-Outer vs. Upper-Inner | 9.15 | 4.12 | 20.33 |
| Upper-Outer vs. Lower-Outer | 22.93 | 8.77 | 59.94 |
| Upper-Outer vs. Lower-Inner | 7.01 | 3.23 | 15.21 |
| Upper-Inner vs. Lower-Outer | 2.50 | 0.94 | 6.65 |
| Upper-Inner vs. Lower-Inner | 0.77 | 0.35 | 1.68 |
| Lower-Outer vs. Lower-Inner | 0.31 | 0.12 | 0.79 |
